# Evaluation of a psychoeducational group intervention for family and friends of youth with borderline personality disorder

**DOI:** 10.1186/s40479-017-0056-6

**Published:** 2017-03-24

**Authors:** Jessie Pearce, Martina Jovev, Carol Hulbert, Ben McKechnie, Louise McCutcheon, Jennifer Betts, Andrew M. Chanen

**Affiliations:** 1 Melbourne School of Psychological Sciences, The University of Melbourne, Melbourne, Australia; 2 Orygen, the National Centre of Excellence in Youth Mental Health, Melbourne, Australia; 3 Centre for Youth Mental Health, The University of Melbourne, Melbourne, Australia; 4 Orygen Youth Health, Northwestern Mental Health, Melbourne, Australia

**Keywords:** Adolescent, Borderline personality disorder, Family, Psychoeducation, Early Intervention, Psychiatry

## Abstract

**Background:**

Despite high levels of burden and distress among families with a member who has borderline personality disorder (BPD), only two BPD specific family psychoeducation groups have been empirically evaluated. Neither of these is designed specifically for the family and friends of young people who are presenting early in the course of BPD. This study aimed to evaluate *Making Sense of Borderline Personality Disorder* (MS-BPD), a three-session, developmentally tailored, manualised psychoeducational group for the family and friends of youth with BPD features.

**Methods:**

The study employed a pre- and post-intervention, repeated measures design. Twenty-three participants completed self-report measures assessing for family burden, psychological distress, and knowledge about personality disorder. Demographic data were collected for the group participants and for their associated young person with BPD. Paired-samples *t*-tests were conducted to evaluate the effect of the MS-BPD intervention on participants’ burden, distress and personality disorder knowledge.

**Results:**

At the completion of session three (day 15), group participants reported significantly decreased subjective burden and increased personality disorder knowledge. Objective burden and distress remained unchanged.

**Conclusions:**

Family and friends of young people with BPD features experienced subjective, but not objective, benefit from attending a brief group-based psychoeducation intervention. Longer follow-up is likely to be required to detect behavioural change. The current findings support proceeding to a randomised controlled trial of MS-BPD.

## Background

Borderline personality disorder (BPD) is a severe mental disorder that usually has its onset in youth (adolescence and emerging adulthood) [1, 2]. BPD is a leading contributor to the burden of disease among the community and is associated with adverse long-term outcomes that include severe and persistent functional disability [3], physical ill health [4] and premature mortality from suicide and natural causes [5, 6].

Caregivers and relatives of adults with BPD experience higher rates of psychological symptoms and distress than the general population [7]. Burden among families with a member with BPD has been reported to be even greater than that associated with other severe mental disorders [8, 9]. This also includes elevated objective and subjective burden, grief, impaired ‘empowerment’ (e.g., difficulties interacting with the mental health service system), and mental health problems, including depression and anxiety [8]. Parents of daughters diagnosed with BPD reported experiencing significant burden in multiple domains that include emotional and physical health problems and marital difficulties [10]. Qualitative studies have also highlighted the chronic and traumatic stress, burden, prolonged hopelessness, shrinking social support, and feelings of grief, guilt and distress experienced by relatives of individuals with BPD [11–14]. One study has even suggested that greater knowledge of BPD is associated with increased family members’ burden, distress and depression [15], raising concerns about the source, accuracy and value of information family members receive abut BPD.

Several interventions have been developed for family members of adults with BPD. These include the McLean psychoeducation program [16], ‘DBT Family Skills Training’ (DBT-FST) [17, 18], ‘Family Connections’ [19], and ‘Staying Connected when Emotions Run High’ [20]. Of these, only the latter two have published evaluations [19–21]. The ‘Family Connections’ program [19] is an offshoot of the DBT-FST program. It consists of 12 weekly sessions led by trained family members providing psychoeducation about BPD, coping and problem-solving skills, family relationship skills, and a support network. In a study of 44 family members of individuals with BPD, participation in the Family Connections program led to reduced burden, decreased grief and an increased perceived capacity to cope at 2 weeks post-program completion, with changes maintained at 6 months post-baseline [19]. A replication study with 55 family members demonstrated a significant increase in perceived capacity to cope and decreases in burden, grief and depression [21]. ‘Staying Connected when Emotions Run High’ [20] is a five-session intervention that is based on a relational treatment model [22] and which focuses on core principles of self-care, keeping calm in distress, setting boundaries, non-directive counselling skills and safety planning. A pilot study of 32 carers reported improvement in well-being and quality of life, in addition to reduced burden, grief and expressed emotion within the family environment [20]. Taken together, these findings suggest that further evaluation of family interventions is warranted.

The establishing of the field of early intervention for BPD and the first wave of clinical trials [23] has seen a specific focus on the early stages of BPD. The onset of a severe mental disorder is an intensely distressing and challenging experience for the young person, their family and friends, and others who care for them. Although some treatments for young people with BPD are more inclusive of families than has been the case historically, the support needs of those closest to these young people, and specific family interventions for this population, have received limited attention.

Despite the emerging support for the effectiveness of family psychoeducation programs for adults with BPD, along with evidence for the effectiveness of family interventions for youth with other severe mental disorders, such as first-episode psychosis [24, 25] or suicidal behaviour [26], there are no published evaluations of family psychoeducation interventions designed specifically for the family and carers of youth with BPD features. Such interventions cannot simply be ‘repurposed’ interventions for adults with BPD because they need to address the needs of early stage disorder and to place BPD in an appropriate development context. Consequently, the current study aimed to evaluate the effectiveness of the *Making Sense of BPD* (MS-BPD) psychoeducation group intervention. It was hypothesised that, upon completion of MS-BPD, group participants would have significantly reduced burden and distress, as well as greater knowledge of personality disorder.

## Methods

### Study design and setting

The current study was a pre- and post-intervention, repeated measures design. The MS-BPD group is a component of the Helping Young People Early (HYPE) program [27], which operates at Orygen Youth Health (OYH), the State Government funded youth mental health service for youth, aged 15–25 years, living in northern and western metropolitan Melbourne, Australia. HYPE is an evidence-based, indicated prevention and early intervention program for youth with BPD features. The program integrates psychologically-informed clinical case management, individual Cognitive Analytic Therapy (CAT; [28]) and general psychiatric care. All family and friends of young people attending HYPE are invited to participate in the MS-BPD group, and the group is open to the family and friends of young people with BPD features who are attending other programs at OYH.

### Intervention

The MS-BPD group intervention integrates concepts from both the HYPE model of care and from CAT, which places emphasis upon BPD as a relational disorder. The group involves three two-hour sessions, conducted over three consecutive weeks (days 1, 8 and 15). It is facilitated by two clinicians and for the final session, a family peer support worker (a family member with lived experience caring for a young person with severe mental health difficulties). Topics covered include the features of personality disorder, diagnosis, causes, treatment, interpersonal skills, relationship patterns, and self-care. These topics are discussed within a youth developmental context.

### Measures

The 15 self-report, true/false BPD items from the Structured Clinical Interview for DSM-IV Axis II Personality Questionnaire (SCID-II-PQ) [29] correspond to the nine DSM-IV BPD diagnostic criteria. A cut point of 13 or higher indicates a probable diagnosis of BPD [30]. Unpublished data from this same study indicate that a score of 9 to 12 indicates sub-syndromal BPD [27]. The SCID-II-PQ BPD items have moderate sensitivity, moderate to high specificity and moderate to high agreement with the BPD diagnosis [30].

The self-report Burden Assessment Scale (BAS) [31] is a 19-item measure of subjective and objective burden of care in families with a severely mentally ill member. Objective burden refers to observable behavioural effects of caregiving such as limitations on personal activities and financial problems, whereas subjective burden includes feelings, attitudes and emotions expressed about caregiving such as shame, stigma and worry [31].

The Kessler Psychological Distress Scale (K-10) [32] is a 10-item self-report questionnaire that assesses for non-specific psychological distress (predominately anxiety and depressive symptoms) over the past 4 weeks. The K-10 has a score range of 10–50, with scores of 10–15 indicating low distress, scores 16–21 moderate distress, 22–29 high distress, and 30–50 very high distress [33].

Knowledge of personality disorders was measured as the sum of three self-report knowledge items taken from the Personality Disorder Knowledge, Attitudes and Skills Questionnaire (PDKASQ) [34, 35]. The original measure was developed as a clinician rated measure of perceived knowledge about PD, thus the wording was changed from “clients” to “people” on two items to make it applicable to family and friends.

### Participants

Over the 10-month recruitment period, 47 family or friends (carers) attended at least one session of the MS-BPD group. Of these, 34 people completed the pre-intervention measures with 29 (85.3%) people completing the post-intervention measures. The majority of group participants attended the MS-BPD within the first 6 months of the young person’s registration with OYH (63.2%). They were derived from the families and friends of 23 OYH clients, the majority of whom were attending the HYPE program (*n* = 14, 73.7%).

There were six dyads of participants (e.g., both parents of a young person with BPD pathology) who attended the MS-BPD group together. In order not to violate assumptions of statistical analyses, only one group participant per young person attending OYH was included in the analyses. For the dyads, the group participant selected was the primary caregiver. If the dyad consisted of two parents, the mother was selected (as mothers were the highest represented group). Thus the final sample comprised 23 group participants who completed both the pre- and post-intervention measures.

Participants were aged between 23 and 66 years (*M* = 49.95 years, *SD* = 9.04 years), including 16 females (69.6%), 6 males (26.1%) and an individual who declined to nominate a gender (4.3%). The majority were parents of a young person attending OYH (15 mothers [65.2%] and 4 fathers [17.4%]), followed by two grandparents (8.7%), one partner (4.3%), and one foster carer (2.9%). The majority attended all three MS-BPD sessions (73.9%).

Additional demographics for the 23 participants included in the study are shown in Table 1.

**Table 1:** Demographic characteristics of MS-BPD group participants (*n* = 23)

Characteristic	Number	Percent
Education level completed		
Secondary	9	39.1%
University	8	34.8%
Trade	3	13.0%
Other/did not state	3	13.0%
Employment status		
Full-time	12	52.2%
Part-time	7	30.4%
Home-maker	2	8.7%
Unemployed	1	4.3%
Did not state	1	4.3%
Marital status		
Married	11	47.8%
Divorced or separated	6	26.1%
Never married	3	13.0%
Other/did not state	3	13.0%
Reported income range per year (AU$)		
$15 600–$31 199	4	17.4%
$31 200–$51 999	5	21.7%
$52 000–$77 999	4	17.4%
$78 000–$103 999	2	8.7%
$104 000 or more	3	13.0%
Declined to answer	5	21.7%

While 23 family and friends participated in the group evaluation, only 19 of the associated young people (82.6%) consented for their demographic and clinical information to be used in this study. These data indicated that MS-BPD group participants were family or friends of predominantly female (84.2%) clients, with a mean age of 17.1 years (*SD* = 1.9 years) and moderate levels of BPD symptomatology (*M* = 11.6, *SD* = 2.2 on the Structured Clinical Interview for DSM-IV Axis II Personality Questionnaire [29] BPD module). Eight young people (42.1%) had SCID-II PQ BPD scores suggesting a possible BPD diagnosis, nine (47.4%) had scores suggesting a possible sub-syndromal BPD diagnosis, and two (10.5%) had scores not suggestive of BPD. Co-occurring (‘comorbid’) mental state and personality disorders were common, including mood disorders (68.4%), anxiety disorders (15.8%), first-episode psychosis (10.5%), other personality disorder traits (10.5%), substance use disorder (5.3%), and an eating disorder (5.3%).

### Procedure

The study was approved by the Melbourne Health Human Research and Ethics Committee. Potential study participants were identified through liaison with clinical staff and referral to the MS-BPD group. The young person (and where appropriate, their parent or guardian) was provided with a Participant Information and Consent Form (PICF) and asked to give written informed consent to use their clinical and referral information, collected as part of their routine clinical care. Potential group participants (family and friends) provided written informed consent and completed the self-report measures prior to the first MS-BPD session. Group participants were able to participate in the study even if their young person did not consent for their case file to be accessed by the researchers. Individuals were able to attend the MS-BPD group if they did not wish to participate in the evaluation. Group participants completed post-intervention measures immediately at the conclusion of the third MS-BPD session (i.e., day 15) or alternatively if they did not attend, participants were contacted via phone and/or post to arrange completion of the post-intervention measures within 1 month of the final session.

### Statistical analysis

Paired-samples *t*-tests were conducted to evaluate the effect of the MS-BPD intervention on participants’ burden, distress and personality disorder knowledge. Three group participants rated an item of the BAS on missed days of employment as not applicable. Scores for these participants were imputed using the Expectation-Maximization (EM) method as this is considered superior to deletion, mean imputation and regression imputation methods [36]. Non-significant Little’s MCAR test pre-intervention, *χ* ^2^ (9) = 15.32, *p* = .08, and post-intervention *χ* ^2^ (9) = 9.98, *p* = .35, indicated the data were missing completely at random [37]. Other missing data (e.g., due to item non-response) was dealt with by pairwise deletion.

## Results

There was a high retention rate, with 85% of group participants who completed the pre-intervention measures also completing the post-intervention measures. The majority of participants completed the post-intervention measures at the conclusion of the final session (*n* = 16, 69.6%). The participants who did not complete the post-intervention measures (*n* = 5) were not significantly different to the rest of the sample, in terms of gender, pre-intervention distress (K-10), pre-intervention burden (BAS) or pre-intervention personality disorder knowledge (PDK) (all *p* > 0.20).

Paired-samples *t*-tests were conducted to evaluate the effect of the MS-BPD intervention on participants’ burden, distress and personality disorder knowledge. Means, standard deviations and *t*-statistics are shown in Table 2.

**Table 2:** Descriptive statistics and t-statistics

Measure	Pre-intervention		Post-intervention		*t*	df	*p*
	Mean	SD	Mean	SD			
BAS	48.19	14.41	44.95	13.73	2.29	22	0.03^a^
BAS - objective	24.96	7.85	24.21	7.79	0.88	22	0.39
BAS - subjective	23.23	7.46	20.74	7.05	2.51	22	0.02^a^
K-10	21.26	8.16	21.37	8.29	−0.08	22	0.94
PDK	8.30	2.57	11.26	2.40	−6.37	22	<0.001^b^

*BAS* burden assessment scale, *K*-*10* kessler psychological distress scale, *PDK* personality disorder knowledge

^a^significant at alpha = 0.05; ^b^significant at alpha = 0.001

Table 2 shows statistically significant differences in pre- to post-intervention scores for overall burden, subjective burden and personality disorder knowledge. Objective burden, and distress remained unchanged. The results demonstrate a small to medium effect size for the overall decrease burden (Cohen’s *d* = .48), a medium effect size for the subjective burden decrease (Cohen’s *d* = .52) and a large effect size for the personality disorder knowledge increase (Cohen’s *d* = 1.33), post-participation in the MS-BPD group intervention.

## Discussion

This is the first study to evaluate the effectiveness of a group psychoeducational intervention that is specifically designed for the family, carers, partners and friends of youth with BPD features. Three major findings emerge from this novel study.

First, the MS-BPD group intervention was found to be associated with a significant reduction in burden of care. Further exploration indicated that participants’ subjective burden (i.e., the feelings, attitudes and emotions expressed about the caregiving experience) significantly decreased, although there were no significant changes in participants’ objective burden (i.e., financial problems and limitations on personal activity). This is important as it suggests that a significant improvement in caregiver’s feelings and attitudes about the caregiving experience can be achieved over a relatively short (15 day) period, using a psychoeducation-based intervention. Moreover, the improvement in subjective burden was reported by caregivers of young people who were relatively new to specialist mental health services (63% were within the first 6 months of registering with OYH, meaning that this was their first experience of evidence-based care for BPD). This period is likely to be a particularly challenging time for families that is marked by acute illness and risk issues, as well as increased distress for families and others. This finding is consistent with previous studies investigating psychoeducation groups for families of adults with BPD [19–21].

In the current study, subjective burden reduced from pre- to post-intervention but there were no changes to objective burden. The absence of significant change in participants’ objective burden in the current study might be related to the duration of the intervention and absence of a follow-up assessment. It is likely that objective burden [31, 38] requires more time and practice to change, compared with subjective burden [39], and could not be captured immediately at the end of the third of three group sessions. The MS-BPD sessions were delivered on days 1, 8 and 15, with the post-intervention assessment occurring immediately at the conclusion of the final session for most participants. In contrast, the Family Connections program [21] ran for 12 weekly sessions and included a follow-up time point at 6 months post-baseline. This might have allowed participants more time to reflect upon and to practice what they had learned and to try to implement successful strategies.

Second, in contrast with the findings of Bailey [20], there were no significant changes in levels of distress among group participants. This disparity might relate to differences in the samples. Bailey studied carers of adults with a BPD diagnosis. The mean length of caregiving relationship in this study was 9.15 years [20], whereas the current study investigated the family and carers of young people with first-presentation BPD who were attending an early intervention program for BPD. As noted, the majority of the MS-BPD group participants attended within the first 6 months of their associated young person being registered at OYH, which might be a particularly chaotic and distressing time for families. The final session of the MS-BPD intervention addresses self-care, led by the family peer support worker. With most participants (69.6%) completing their post-intervention measures at the conclusion of this session, there was inadequate time for participants to enact these self-care strategies, which might have been beneficial in reducing their distress.

Third, participants in the MS-BPD group reported increased knowledge of personality disorders over the study period. None of the previous studies evaluating family psychoeducation interventions for adults with BPD [19–21] have examined personality disorder knowledge and therefore, the current study extends the limited literature in the field. Of note, a previous study found that greater knowledge of BPD among family members was associated with increased burden, distress and depressive symptoms [15]. The authors suggested that inaccurate information about BPD, which was common at the time of the study, might have contributed to negative outcomes for families. The understanding of BPD and evidence-based treatments for BPD has improved over recent times among adults [40] and youth [23], therefore, it is possible that our current understanding of the treatability of BPD allows for realistic hope. The present findings suggests that by presenting information in an appropriately sensitive manner to families in a psychoeducation group setting, it is possible to increase knowledge without resulting in negative outcomes. This increases confidence that intervening early for youth with BPD pathology and their families will lead to positive outcomes. It is noteworthy that the selected PDKASQ items assess for participants’ perception of their knowledge (rather than actual knowledge). While sufficient for the purpose of this preliminary study, future studies should assess for actual knowledge of BPD.

This preliminary study has several limitations. The absence of a control condition means that changes in participants’ burden and knowledge cannot be solely attributed to the MS-BPD intervention. The study involved a pre- and post- repeated measures analysis with no follow up time points and therefore, it is unknown whether improvements were maintained or whether further improvement occurred over time. In addition, despite the manualised nature of the MS-BPD intervention, the study did not account for potential group clustering and assumed that all MS-BPD groups were the same. In support of this assumption, a researcher (JP) observed all sessions to ensure manual adherence. A final methodological limitation is that diagnostic information about the young person attending OYH was taken from their MS-BPD referral form, rather than formal diagnostic assessment.

Notwithstanding these limitations, this is the first study to evaluate the effectiveness of a group intervention designed specifically to provide BPD psychoeducation within a youth developmental context for family members, carers, partners and friends of youth with BPD features. This is important because reducing the time between illness onset and the family attending a psychoeducation group is likely to be central to increasing accurate knowledge and reducing perceived stigma and burden. In previous studies, the average time from the onset of BPD to attending a family psychoeducation group ranged from 7.7 [19] to 13.7 years [21]. This is likely to be shorter in the current study, given the clients’ mean age of 17 years and the HYPE early intervention model, which welcomes participants with their first presentation for care of BPD. Furthermore, the current findings show that a significant improvement in family and friends’ subjective burden can be achieved using a brief psychoeducation-based group intervention, implemented over 15 days, within the first 6 months of service entry. This has potential to improve engagement and outcome for young people and their families.

## Conclusions

These preliminary findings suggest that MS-BPD is a time effective and resource-appropriate way to deliver information to families and friends in a busy, publicly funded youth mental health service. MS-BPD yields improvements in subjective burden and knowledge of BPD over a relatively short time period. The current findings suggest that it would be worthwhile to proceed to a randomised controlled trial. Such a trial should investigate the effectiveness of the MS-BPD group, compared with provision of psychoeducational material in a non-group format. The follow-up assessment should be delayed to allow time for participants to implement skills and knowledge learned during the program. Outcome measures should include family burden, knowledge of BPD, coping and distress.

## Abbreviations

BAS: Burden assessment scale
BPD: Borderline personality disorder
CAT: Cognitive analytic therapy
DBT FST: Dialectical behaviour therapy family skills training
EM: Expectation-maximization
HYPE: Helping young people early
K-10: Kessler psychological distress scale
MS-BPD: Making sense of borderline personality disorder
OYH: Orygen youth health
PDKASQ: Personality disorder knowledge attitudes and skills questionnaire
PICF: Participant information and consent form
SCID-II PQ: Structured clinical interview for DSM-IV axis II personality questionnaire
